# An Integrated Approach to Determine the Boundaries of the Azaphilone Pigment Biosynthetic Gene Cluster of *Monascus ruber* M7 Grown on Potato Dextrose Agar

**DOI:** 10.3389/fmicb.2021.680629

**Published:** 2021-06-16

**Authors:** Qingpei Liu, Siyu Zhong, Xinrui Wang, Shuaibiao Gao, Xiaolong Yang, Fusheng Chen, István Molnár

**Affiliations:** ^1^The Modernization Engineering Technology Research Center of Ethnic Minority Medicine of Hubei Province, School of Pharmaceutical Sciences, South-Central University for Nationalities, Wuhan, China; ^2^Southwest Center for Natural Products Research, The University of Arizona, Tucson, AZ, United States; ^3^Hubei International Scientific and Technological Cooperation Base of Traditional Fermented Foods, Huazhong Agricultural University, Wuhan, China; ^4^College of Food Science and Technology, Huazhong Agricultural University, Wuhan, China

**Keywords:** *Monascus* azaphilone pigment, gene cluster boundary, comparative genomics, transcription analysis, gene knockout

## Abstract

*Monascus-*type azaphilone pigments (MonAzPs) are produced in multi-thousand ton quantities each year and used as food colorants and nutraceuticals in East Asia. Several groups, including ours, described MonAzPs biosynthesis as a highly complex pathway with many branch points, affording more than 110 MonAzP congeners in a small group of fungi in the Eurotiales order. MonAzPs biosynthetic gene clusters (BGCs) are also very complex and mosaic-like, with some genes involved in more than one pathway, while other genes playing no apparent role in MonAzPs production. Due to this complexity, MonAzPs BGCs have been delimited differently in various fungi. Since most of these predictions rely primarily on bioinformatic analyses, it is possible that genes immediately outside the currently predicted BGC borders are also involved, especially those whose function cannot be predicted from sequence similarities alone. Conversely, some peripheral genes presumed to be part of the BGC may in fact lay outside the boundaries. This study uses a combination of computational and transcriptional analyses to predict the extent of the MonAzPs BGC in *Monascus ruber* M7. Gene knockouts and analysis of MonAzPs production of the mutants are then used to validate the prediction, revealing that the BGC consists of 16 genes, extending from *mrpigA* to *mrpigP*. We further predict that two strains of *Talaromyces marneffei*, ATCC 18224 and PM1, encode an orthologous but non-syntenic MonAzPs BGC with 14 genes. This work highlights the need to use comprehensive, integrated approaches for the more precise determination of secondary metabolite BGC boundaries.

## Introduction

*Monascus-*type azaphilone pigments (MonAzPs) are a complex mixture of secondary metabolites (SMs) with a tricyclic azaphilone scaffold, produced by a few fungal genera in the Eurotiales order such as *Monascus* and *Talaromyces* spp. MonAzPs are traditionally classified as red, orange, and yellow pigments based on their absorbance maxima (Feng et al., 2012). To the best of our knowledge, more than 110 MonAzPs components have been identified from various fungi (Chen et al., 2019). As colorants, MonAzPs have been widely used in various food products for centuries, especially in Southeast Asian countries (Chen et al., 2015). Moreover, MonAzPs also possess wide-ranging biological activities such as preventing hypertension (Lee and Pan, 2012a), lowering cholesterol levels (Lee et al., 2010), causing hypolipidemic effects (Lee and Pan, 2012b), and displaying anti-obesity (Choe et al., 2012) and anti-tumor activities (Akihisa et al., 2005).

Investigations of the MonAzPs biosynthetic pathway started in the 1960s (Birch et al., 1962; Kurono et al., 1963; Hadfield et al., 1967). With the advent of fungal genome sequencing, several groups, including ours, proposed a unified MonAzPs biosynthetic process active in various *Monascus* species and strains that differ in their azaphilone pigment and citrinin productivities (He and Cox, 2016; Chen et al., 2019). This pathway was found to consist of a trunk pathway with many biosynthetic branches that use enzymes with substrate- and product flexibility. It was also seen to utilize adventitious biochemical or chemical transformations, and to incorporate some still not well characterized biosynthetic steps (Chen et al., 2019). Functional studies on the biosynthesis of MonAzPs utilized a combination of targeted gene knockouts, heterologous gene expression, and *in vitro* chemical and enzymatic reactions (Balakrishnan et al., 2013, 2014a,b,c, 2015, 2017a,b,c; Xie et al., 2013; Liu et al., 2014; Liu J. et al., 2016; Chen et al., 2017; Liang et al., 2018; Li et al., 2020). These studies also revealed that the MonAzPs biosynthetic gene cluster (BGC) in *Monascus ruber* M7 also encodes the biosynthesis of monasones, anthraquinone-type SMs with antibacterial activities (Li et al., 2020).

The functional and structural complexities of MonAzPs BGCs and biosynthetic processes, and their variability among different fungi make it difficult to predict the extent of these BGCs, especially when relying only on routine sequence similarity searches to draw cluster boundaries. It remains possible that genes outside the currently predicted boundaries of the BGC are also involved in MonAzPs production, especially when their functions cannot be easily predicted from the similarities of their encoded proteins alone. Conversely, it is also possible that bioinformatic methods over-estimate the extent of MonAzPs BGCs, and include genes in the predicted clusters that have in fact no role in pigment biosynthesis. Such prediction mistakes may undermine biosynthetic proposals by omitting important genes or including spurious ones, thus highlighting the need for more comprehensive prediction workflows to delimit BGC boundaries.

The carbon skeletons of SMs are often synthesized by “core” enzymes such as polyketide synthases (PKSs) and non-ribosomal peptide synthetases (NRPSs). Several widely used software tools that predict fungal SM BGCs, including SMURF^1^ and antiSMASH^2^ detect the genes encoding such core enzymes, and anchor the predicted BGCs around these genes (Khaldi et al., 2010; Blin et al., 2019). However, these very useful software tools are not particularly well suited to define BGC boundaries. To address this need, Takeda et al. (2014) devised a novel comparative genomics method to predict the extent of SM BGCs by searching for gene similarities in genome sequence assemblies, and by evaluating the presence of similar genes even in non-syntenic blocks. This method made it possible to better identify known SM BGCs featuring core genes, and some even without such anchors, in the genome sequences of 10 filamentous fungi (Takeda et al., 2014).

The biosynthesis of fungal SMs is governed by a hierarchical regulatory network that often involves pathway-specific regulators (Lyu et al., 2020). Some pathway-specific regulators control the transcription of all the genes involved in the production of a given SM, while others regulate only a key subset of the structural genes. For example, the transcription factor Sol4 governs all six biosynthetic genes in the BGC of solanapyrone, a polyketide-derived phytotoxic SM from the fungus *Ascochyta rabiei* (Kim et al., 2015). Such pathway-specific regulators may also be exploited for cluster boundary predictions. Thus, the boundaries of the BGC for azanigerone A, an azaphilone pigment from *Aspergillus niger*, were predicted using RT-PCR analysis in a strain with an activated pathway-specific positive regulator (Zabala et al., 2012). In contrast, the expression of only some key structural genes is modulated by the pathway-specific regulators for apicidin, a histone deacetylase inhibitor, fusaric acid, a mycotoxin produced by fusaria, and sterigmatocystin, a carcinogenic mycotoxin produced by aspergilli (Jeon et al., 2011; Studt et al., 2016; Wiemann et al., 2018). Finally, pathway-specific regulators may also modulate the expression of genes with no obvious function in SM biosynthesis, resistance or export, as is the case with the fusarin C BGC in *Fusarium fujikuroi* (Niehaus et al., 2013). Therefore, transcription analysis that relies solely on the differential expression of genes governed by a pathway-specific regulator to predict SM BGC boundaries may also omit important genes, or overestimate BGC size due to pleiotropic effects, or the extent of euchromatic regions.

In the current study, we determined the boundaries of the MonAzPs BGC of *Monascus ruber* M7 by an integrated approach. We compared a 100 kb stretch of the *M. ruber* M7 genome flanking the MonAzPs PKS gene *mrpigA*, to the genome sequences of eight other species of filamentous fungi. Next, we conducted a differential transcriptomic analysis of the MonAzPs BGC in *M. ruber* M7 and its knockout mutant deficient in the pathway-specific regulator MrPigB. Finally, we knocked out genes at the predicted BGC boundaries, and compared the MonAzPs metabolic profiles of the resulting strains to that of the wild-type strain. This work provides a more accurate prediction of the extent of the MonAzPs BGC in *M. ruber* M7, and by extension in other *Monascus-*type azaphilone pigment producer fungi. It also exemplifies a workflow to predict, with higher confidence, the boundaries of similarly complex SM BGCs in other fungi.

## Materials and Methods

### Fungal Strains, Culture Conditions, and DNA Extraction

The wild-type strain *Monascus ruber* M7 (CCAM 070120, Culture Collection of the State Key Laboratory of Agricultural Microbiology, China Center for Type Culture Collection, Wuhan, China) (Chen and Hu, 2005; Liu et al., 2014; Liu J. et al., 2016; Chen et al., 2017, 2019; Li et al., 2020) and its derivatives used in this study are listed in Table 1. For the generation of the Δ*mrpigAup1*, Δ*mrpigAup2*, Δ*mrpigPdown1*, and Δ*mrpigPdown2* strains, initial transformants were selected on potato dextrose agar (PDA) medium containing 30 μg/mL hygromycin B (Sigma-Aldrich, Shanghai, China) at 28°C. For phenotypic characterization, all the tested strains were cultivated in triplicates on PDA plates at 28°C for 10 d. Fungal genomic DNA was isolated from mycelia grown on cellophane membranes covering PDA plates, using the cetyltrimethylammonium bromide (CTAB) method (Shao et al., 2009).

**TABLE 1:** *Monascus ruber* strains used in this study.

Strain	Parent	Genotype	References
*M. ruber* M7	–	Wild-type	Chen and Hu, 2005
Δ*mrpigAup2*	*M. ruber* M7	Δ*mrpigAup2*::*hph*	This study
Δ*mrpigAup1*	*M. ruber* M7	Δ*mrpigAup1*::*hph*	This study
Δ*mrpigA*	*M. ruber* M7	Δ*mrpigA*::*hph*	Xie et al., 2015
Δ*mrpigB* (Δ*pigR*)	*M. ruber* M7	Δ*mrpigB*:*hph*	Xie et al., 2013
Δ*mrpigO*	*M. ruber* M7	Δ*mrpigO*::*hph*	Chen et al., 2017
Δ*mrpigP*	*M. ruber* M7	Δ*mrpigP*::*hph*	Chen et al., 2017
Δ*mrpigPdown1*	*M. ruber* M7	Δ*mrpigPdown1*::*hph*	This study
Δ*mrpigPdown2*	*M. ruber* M7	Δ*mrpigPdown2*::*hph*	This study

### Bioinformatic Methods

**FIGURE 1:**
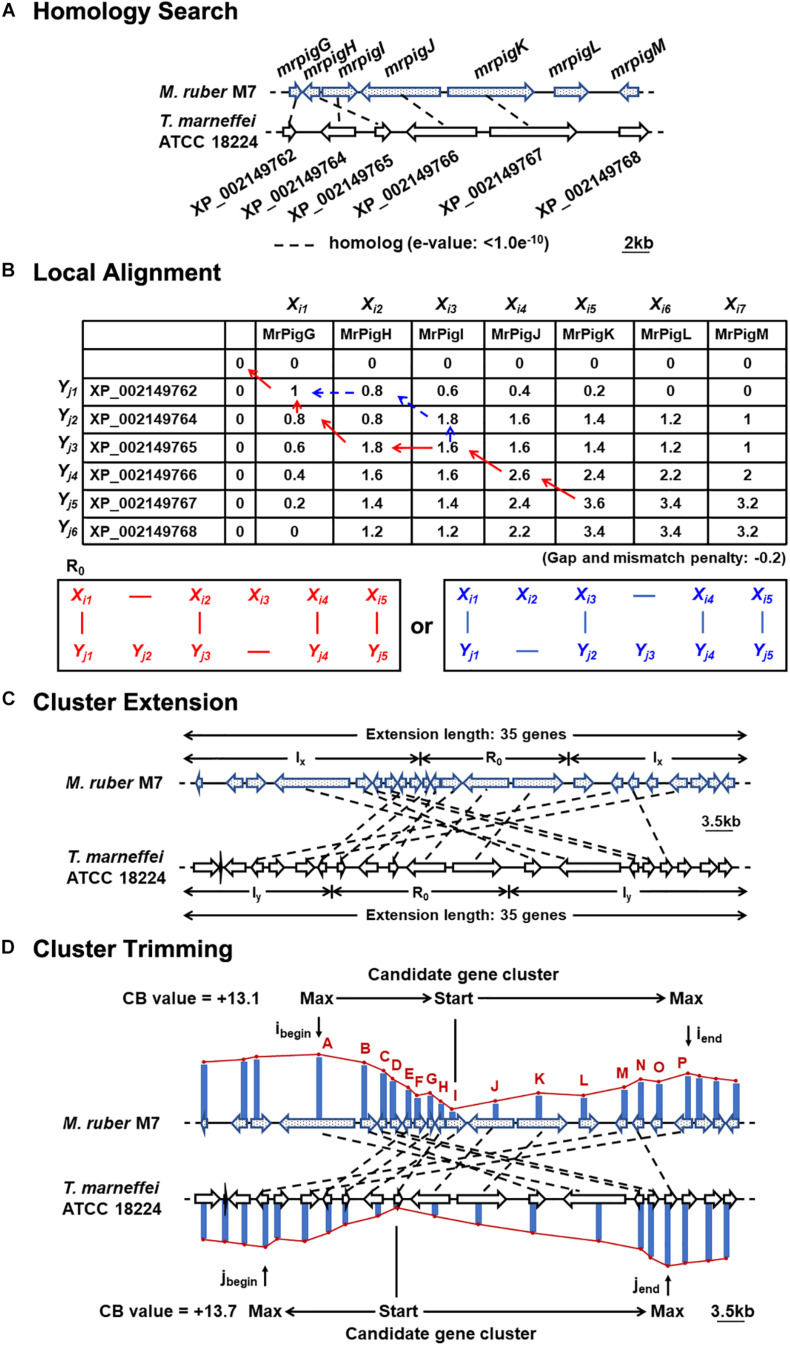
Motif-independent comparative genomic prediction of the MonAzPs BGC boundaries. **(A)** Homology search against the deduced proteome of *T. marneffei* ATCC 18224 using the MonAzPs biosynthesis-related proteins of *M. ruber* M7 as the bait. *Dashed lines*, gene pairs encoding homologous proteins (*e* < 1.0e^–10^ for the encoded proteins). Due to mis-annotation, XP_002149763 is described in NCBI as a separate gene; however, this nucleotide sequence is in fact part of the XP_002149764 gene. **(B)** Local protein sequence alignment using the Smith-Waterman algorithm. Pairs of contiguous genes encoding MrPigG to MrPigK in *M. ruber* M7 and from XP_002149762 to XP_002149767 in *T. marneffei* ATCC 18224 form the seed region (R_0_) for predicting the MonAzPs BGC. SW scores shown were calculated as described (Takeda et al., 2014). **(C)** Extension of the gene cluster. The seed region (R_0_) was extended to include a total of 35 genes (Takeda et al., 2014). The symbols I_*x*_ and I_*y*_ represent the stretches of genes added to the seed region in the *M. ruber* M7 and the *T. marneffei* ATCC 18224 genomes, respectively. **(D)** Trimming of the BGC boundaries. *i*_*begin*_ and *i*_*end*_, the locations of the genes at the beginning and end, respectively, of the MonAzPs gene cluster in *M. ruber* M7; *j*_*begin*_ and *j*_*end*_, the corresponding gene locations in *T. marneffei* ATCC 18224. *CB values* are the maximum cumulative SW scores of the predicted BGCs with the upstream and the downstream boundaries indicated (Takeda et al., 2014).

The nucleotide and deduced amino acid sequences of the genomes of nine strains of filamentous fungi, including *M. ruber* M7, were retrieved from the Broad Institute^3^ and GenBank^4^, as shown in Supplementary Table 1. To predict the MonAzPs gene cluster boundaries, the predicted proteomes encoded by these genome sequences were subjected to comprehensive pairwise comparisons (Takeda et al., 2014). Briefly, pairwise similarities among the deduced proteome of *M. ruber* M7 and the other species were determined first, to detect orthologous, co-located genes that may form a BGC “seed” region (*e* < 1.0e^–10^; gap penalty: −0.2; and mismatch penalty: −0.2; Figures 1A,B). In the second step, the seed region was extended and the boundaries of the extended BGC were trimmed (extension length: 35 genes; negative penalty: −0.3; Figures 1C,D), as previously described (Takeda et al., 2014).

100–kb sequences flanking the MonAzPs polyketide synthase MrPigA (Xie et al., 2015) in the genome of *M. ruber* M7 were submitted to antiSMASH 5.0 (Blin et al., 2019), and PRISM (Skinnider et al., 2020) for SM gene cluster characterization. The MIBiG 2.0 curated repository for SM BGCs was also considered (Kautsar et al., 2020). The SMURF^5^ and CASSIS servers^6^ could not be accessed, or returned uncharacterized errors. The amino acid sequences encoded by the genes were deduced using FGENESH^7^, and analyzed using Pfam 27.0^8^. Similarities of the deduced amino acid sequence were analyzed using BLASTP^9^.

### Quantitative Reverse Transcription PCR (RT-qPCR) Analysis

The wild-type *M. ruber* M7 and the pathway-specific regulator knockout strain Δ*mrpigB* were cultured in PDB medium with shaking at 180 rpm for 6 days at 28°C (start of the active production phase for MonAzPs). RT-qPCR was performed by Wuhan Goodbio Technology Co., Ltd. (Wuhan, Hubei, China) as described by Liu et al. (2014). For both the wild-type and Δ*mrpigB* strains, three biological replicates were analyzed, and for each sample, three technical replicates for each targeted gene were performed. GADPH was used as the reference gene, and the relative expression fold-change was calculated using the comparative CT method. Significance analysis was performed using the one-way ANOVA test and significance level was set as 0.05. The used primers are listed in Supplementary Table 2.

### Gene Knockouts

*M. ruber* M7 knockout strains Δ*mrpigA*, Δ*mrpigO*, and Δ*mrpigP* have been described (Xie et al., 2013, 2015; Chen et al., 2017; Li et al., 2020). For the deletion of the *mrpigAup1* gene, a gene disruption cassette carrying the hygromycin B resistance gene (*hph*) flanked by targeting arms (TA) derived from the 5′ or 3′regions of *mrpigAup1*, respectively, was prepared using the double-joint PCR method (Yu et al., 2004). Briefly, the 5′ and 3′flanking regions (910 and 893 bp, respectively) of the *mrpigAup1* gene were amplified with the primer pairs P1–P2 and P3–P4, respectively. The 2.1 kb *hph* cassette was amplified from plasmid pSKH (He et al., 2013) with the primer pair P7–P8. The PCR products were purified with a TransGen gel purification kit (TransGen, Beijing, China), the three amplicons were mixed at a 1:1:2 molar ratio, then fused by PCR using primer pair P1–P4. The fused PCR product was purified, cloned into pMD19–T (Takara, Dalian, Japan), and confirmed by sequencing. The disruption cassette was then transferred from the resulting plasmid into the *Kpn*I and *Xba*I sites of pCAMBIA3300 (He et al., 2013) to generate plasmid pCPIGAUP1. Analogous strategies were used to generate the pCPIGAUP2, pCPIGPDOWN1, and pCPIGPDOWN2 plasmids for the deletion of *mrpigAup2*, *mrpigPdown1*, and *mrpigPdown2*, respectively, using primers listed in Supplementary Table 3.

The gene disruption plasmids were individually transformed into *Agrobacterium tumefaciens* EHA105 (Hood et al., 1993) using the freeze-thaw method (Yu et al., 2003), and used for the transformation of *M. ruber* M7 to yield the gene knockout strains Δ*mrpigAup1*, Δ*mrpigAup2*, Δ*mrpigPdown1*, and Δ*mrpigPdown2*, using methods described previously (Shao et al., 2009; Wang et al., 2011). Gene knockouts and the absence of the wild-type allele in the mutants were confirmed by PCR and end sequencing of the resulting amplicons.

### MonAzPs Analysis

Freshly harvested spores (5 × 10^4^) of representative isolates of the gene knockout strains, Δ*mrpigAup1*, Δ*mrpigAup2*, Δ*mrpigA*, Δ*mrpigO*, Δ*mrpigP*, Δ*mrpigPdown1*, Δ*mrpigPdown2*, and the wild-type strain *M. ruber* M7 were spread on cellophane membranes on PDA plates, and cultivated at 28°C for 10 days. The mycelia were harvested by scraping from the membranes, freeze-dried, and ground in a mortar with a pestle under liquid nitrogen. The mycelia powder (0.05 g) was suspended in 1.5 mL methanol, incubated at 65°C for 1 h, then centrifuged at 10,000 × g for 10 min to collect the supernatant for analysis. HPLC was performed following the method described by Liu Q. et al. (2016) on a Waters system fitted with an Inertsil ODS–3 C18 column (250 × 4.6 mm, 5.0 μm, GL Sciences). The mobile phases consisted of water (A), acetonitrile (B), and 0.5% phosphoric acid in water (C). The flow rate was kept at 0.8 mL/min. The system was run with the following gradient program: from 40 to 30% A for 3 min, from 30 to 5% A for 22 min, 5% A for 5 min, from 5 to 40% A for 1 min, and 40% A for 5 min. C was kept constant at 5% throughout the program. Absorbance was monitored with a 2487 UV/Vis Detector (Waters) at 190- to 700-nm wavelength. Metabolites were identified based on comparison to authentic standards (Chen et al., 2017, 2019).

### Accession Numbers

The genes *mrpigAup1*, *mrpigAup2*, *mrpigPdown1*, and *mrpigPdown2* of *M. ruber* M7 have been assigned GenBank accessions MH729876, MW557663, KC561931, and MW557664, respectively.

## Results

### Bioinformatic Prediction of the MonAzPs BGC Boundaries

**FIGURE 2:**
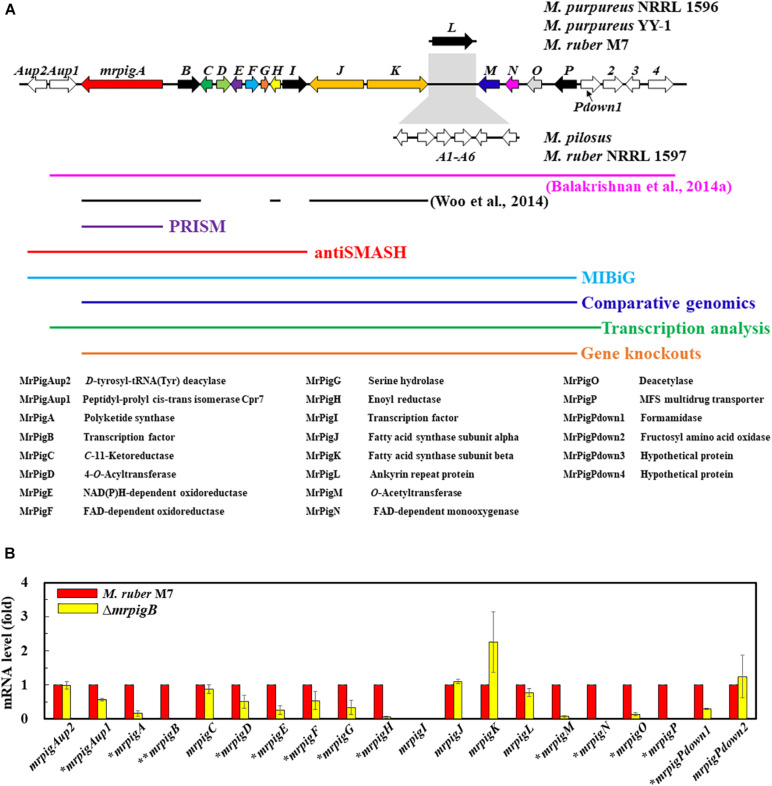
Bioinformatic and transcriptomic analysis of the MonAzPs BGC. **(A)** Gene map of the MonAzPs locus and the extent of the MonAzPs BGC in different fungi according to different authors and different bioinformatic or experimental assignment methods. The predicted functions of the proteins encoded in the MonAzPs locus are also listed. **(B)** RT-qPCR analysis of the genes in the MonAzPs locus of the wild-type *M. ruber* M7 strain and its Δ*mrpigB* derivative, measured under identical cultication conditions. Gene expression levels in the wild-type M7 strain are taken as the basis of comparison, with the means and standard deviation calculated from measurements in three biological replicates for each strain. Statistically significant differences (*p* < 0.05) in expression levels are indicated by *stars*.

MonAzPs BGCs display two characteristically different architectures in the sequenced genomes of *Monascus* spp. (Chen et al., 2017). In *M. ruber* M7 and *M. purpureus* strains NRRL 1596 and YY–1, the MonAzPs cluster is interrupted by the *pigL* gene that encodes an ankyrin repeat protein with no discernible function in MonAzPs biosynthesis (Figure 2A; Chen et al., 2017). In *M. ruber* NRLL 1597 and *M. pilosus*, the *pigL* gene is replaced by a six-gene sub-cluster encoding proteins with putative transport or regulatory functions, but none of these proteins are predicted to be necessary for MonAzPs biosynthesis (Balakrishnan et al., 2014a; Chen et al., 2017). Since the insertion of this six-gene sub-cluster would easily confuse bioinformatic methods for BGC boundary prediction, we concentrated on the near-identical genomic loci of the *M. ruber* M7 and the *M. purpureus* strains.

The MonAzPs BGC in *M. purpureus* was originally delineated by Balakrishnan et al. (2014a) by manually comparing *Monascus* spp. genomic loci, and described to include 21 genes over 60 kb, extending from *mpp–1* (ortholog of *mrpigAup1* in *M. ruber* M7) to *mpp–14* (ortholog of *mrpigPdown4* in M7) (Figure 2A). The MIBiG (Minimum Information about a Biosynthetic Gene Cluster, Kautsar et al., 2020), a curated repository of BGCs, describes the *M. pilosus* MonAzPs cluster to extend from AGN71602 to AGN71625, corresponding to *mrpigAup2* to *mrpigP* on the *M. ruber* M7 genome, but inclusive of the six-gene sub-cluster with no role in MonAzPs biosynthesis (Figure 2A). In contrast, antiSMASH (Blin et al., 2019), the computational method most frequently used to define SM BGCs, delineated the cluster to include only 11 genes (*mrpigAup2* to *mrpigI*), spanning approximately 30 kb in the *M. ruber* M7 or the two *M. purpureus* genomes (Figure 2A). PRISM (Skinnider et al., 2020), another SM BGC prediction tool^10^, identified only *mrpigA* as the sole constituent of the cluster. Unfortunately, neither SMURF (Khaldi et al., 2010) nor CASSIS (Wolf et al., 2016) provided useful output for our comparisons, due to persistent problems with the webservers. Considering these radically different cluster boundary predictions, we sought a more definite workflow to delimit the complex MonAzPs BGC.

To do this, we first considered the motif-independent comparative genomics method described by Takeda et al. (2014). In a preliminary step, we compared a 100-kb stretch bracketing the MonAzPs polyketide synthase *mrpigA* (Xie et al., 2015) on the *M. ruber* M7 genome with the genome sequences of eight other species of filamentous fungi from the Eurotiales order (Supplementary Table 1). We have not included the genomes of other *Monascus* spp. in this comparison, considering that the extended MonAzPs loci in these genomes are syntenic apart from the presence of *pigL* vs. the six-gene sub-cluster, and that the encoded protein sequences are near-identical (95–100% identity) (Chen et al., 2017). Thus, these loci would have furnished no useful information for our analysis. The preliminary comparison of the *M. ruber* M7 genes identified the highest similarities to the genome of *Talaromyces (Penicillium) marneffei* ATCC 18224, a thermally dimorphic opportunistic fungal pathogen endemic in Southeast Asia and associated with immunocompromised individuals. Thus, we used the genome sequence of this fungus as the comparator for our subsequent analysis. Using the optimized parameters (Takeda et al., 2014), we assigned the seed region (*R*_0_) of the MonAzPs cluster to include *mrpigG* to *mrpigK* (XP_002149762 to XP_002149767 in the *T. marneffei* ATCC 18224 genome, Figures 1A,B). To extend the seed region, 15 genes bracketing *R*_0_ were added to each end (regions *I*_*x*_, Figure 1C), yielding a 35–gene candidate BGC (Takeda et al., 2014). Finally, the boundaries of the candidate cluster were trimmed at both ends, based on the local maxima of the combined scores for the member genes (Figure 1D). This analysis predicted that the MonAzPs BGC extends from *mrpigA* to *mrpigP* in the *M. ruber* M7 genome (Figure 2A).

### Transcriptional Analysis of the MonAzPs Locus in *M. ruber* M7

Analysis of mutants of the pathway-specific Zn(II)_2_Cys_6_ regulators has proven to be useful in demarcating the ends of SM BGCs (Zabala et al., 2012; Wiemann et al., 2013a). In *M. ruber* M7, MonAzPs biosynthesis is governed by the pathway-specific regulator MrPigB (PigR1 in *M. pilosus*) (Balakrishnan et al., 2013; Xie et al., 2013). To substantiate the bioinformatic prediction of the boundaries of the MonAzPs BGC, we used quantitative reverse transcription PCR (RT-qPCR) to compare the transcription levels of the 20 genes flanking *mrpigA* (i.e., *mrpigAup2* to *mrpigPdown2*) at the start of MonAzPs accumulation in the wild-type *M. ruber* M7 with those of the same genes in the Δ*mrpigB* mutant. As shown in Figure 2B, all these genes were expressed in the wild-type strain, apart from *mrpigI* that encodes a transcription factor with no apparent role in MonAzPs biosynthesis (Chen et al., 2017). The expression of *mrpigAup2* and *mrpigPdown2*, the two genes at the edges of the MonAzPs locus were not affected by the deletion of *mrpigB*, indicating that these genes may lay outside the boundaries of the BGC, as expected from the comparative genomic analysis (Figure 1). The expression of six MonAzPs genes (*mrpigA, mrpigH*, and *mrpigM-mrpigP*) was almost completely abolished in the Δ*mrpigB* strain, as observed earlier (Li et al., 2020). The transcription of a further six genes was also moderately downregulated (*p* < 0.05). These included *mrpigD-mrpigE*, all with established roles in MonAzPs biosynthesis. However, the expression of *mrpigAup1* and *mrpigPdown1* were also significantly reduced in the Δ*mrpigB* strain, indicating that the transcription of these genes with no known roles in MonAzPs biosynthesis is, nevertheless, activated by MrPigB (Figure 2B). These two genes were predicted to lay outside the MonAzPs BGC by comparative genomic analysis (Figure 1).

The transcriptional analysis is further complicated by the fact that the expression of four genes well inside the MonAzPs locus (*mrpigC*, *mrpigJ*, *mrpigK*, and *mrpigL*) were not affected by the deletion of *mrpigB*. Among these, the putative ankyrin repeat protein MrPigL plays no role in the production of MonAzPs (Chen et al., 2017). However, *mrpigC* encodes the C11–ketoreductase, *mrpigJ*, and *mrpigK* encode the two subunits of the fatty acid synthase that are all necessary for MonAzPs biosynthesis (Chen et al., 2017). Thus, while *mrpigC*, *mrpigJ*, and *mrpigK* must be part of the MonAzPs BGC, their expression is not directly controlled by MrPigB—a consequence of the complex, mosaic-like structure of this BGC (Li et al., 2020).

Combining the comparative genomic prediction with the transcriptomic analysis, *mrpigAup2*, and *mrpigPdown2* are not likely to be part of the MonAzPs BGC, while the cluster can be confidently predicted to extend at least from *mrpigA* to *mrpigP*. However, the status of *mrpigAup1* and *mrpigPdown1* remains ambiguous in light of the disagreement of the bioinformatic prediction and the transcription data (Figure 1 vs. Figure 2).

### Knockout of the Genes at the Predicted Boundaries of the MonAzPs BGC

**FIGURE 3:**
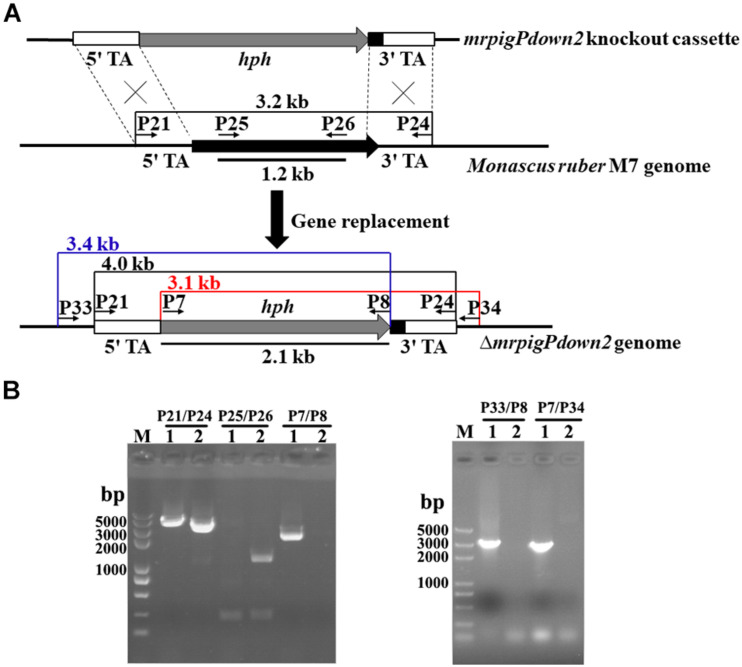
Knockout of the *mrpigPdown2* gene in *M. ruber* M7. **(A)** Schematic representation of the gene knockout strategy yielding the Δ*mrpigPdown2* strain. The primers, and the sizes of the corresponding PCR amplicons used to verify the gene knockout event are indicated. **(B)** Confirmation of the gene deletion event using PCR. *Lane 1*, a representative isolate of the Δ*mrpigPdown2* strain; *Lane 2*, the wild-type strain *M. ruber* M7.

Since the comparative genomic and transcriptomic analyses still did not provide a definitive answer to the extent of the MonAzPs BGC, we sought additional functional proof for the roles of the genes at the proposed BGC boundaries. Thus, we created four new gene knockout strains, affecting the genes that lay at/beyond the border of the MonAzPs BGC in *M. ruber* M7. Five hygromycin B resistant, putative *mrpigPdown2* knockout isolates were obtained by ATMT (*Agrobacterium tumefaciens-*mediated transformation). The putative disruptants and the wild-type control were characterized by PCR analysis, using primer pairs specific for the *hygR* transgene (P7–P8); the *mrpigPdown2* target gene (P25–P26); the gene replacement cassette (P21–P24); and the gene replacement locus (P33–P8 and P7–P34, Figure 3). All five putative disruptants yielded amplicons whose sizes and nucleotide sequences were consistent with the integration of the *hygR* gene into the targeted locus. At the same time, these isolates failed to provide amplicons specific for the target gene, consistent with the loss of *mrpigPdown2* from the genome. The gene knockouts were validated using analogous procedures for the *mrpigAup1* (six verified isolates; Supplementary Figure 1), *mrpigAup2* (two verified isolates; Supplementary Figure 2), and the *mrpigPdown1* genes (three verified isolates; Supplementary Figure 3).

### Phenotypic and MonAzPs Production Analysis Confirms the BGC Boundaries

**FIGURE 4:**
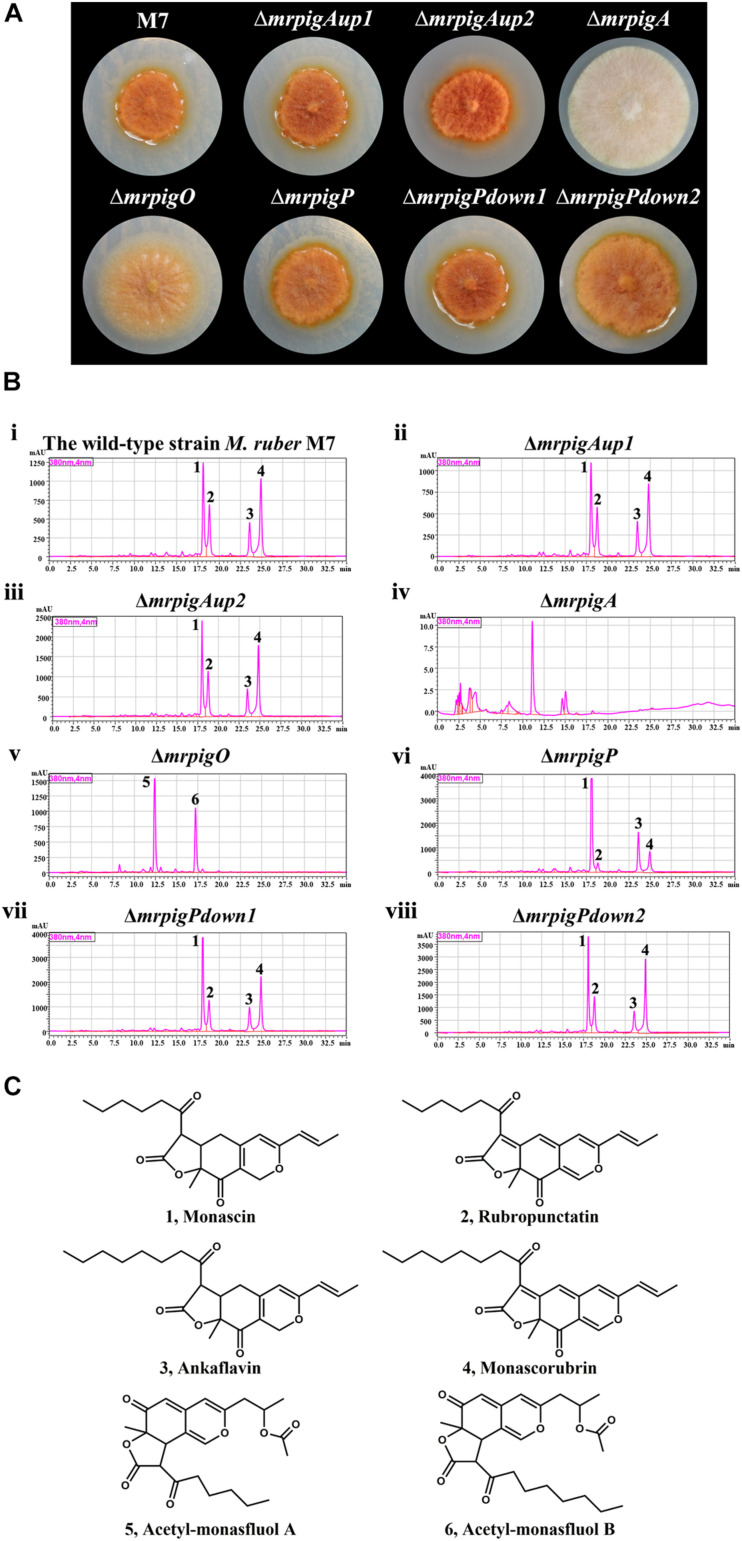
Colony morphology and MonAzPs production of *M. ruber* strains. **(A)** Representative colonies of the indicated *M. ruber* strains, grown at 28°C for 10 d on PDA plates. **(B)** HPLC traces (PDA, 380 nm) of crude extracts of the cultures of the indicated strains. Unlabeled peaks are unidentified *M. ruber* metabolites not related to MonAzPs. **(C)** Structures of MonAzPs.

We cultivated the wild-type strain *M. ruber* M7 and representative isolates of the knockout strains Δ*mrpigAup1*, Δ*mrpigAup2*, Δ*mrpigPdown1*, and Δ*mrpigPdown2* together with the previously isolated disruptant strains Δ*mrpigA*, Δ*mrpigO*, Δ*mrpigP*, and analyzed their colony phenotypes and MonAzPs production (Figure 4). The wild-type *M. ruber* M7 strain is able to produce several major MonAzPs congeners (Chen et al., 2017). Its product spectrum is determined by the culture conditions, including the ingredients of the culture media and the cultivation time. The growth of strain M7 on PDA plates at 28°C for 10 days afforded colonies of an intense orange color, corresponding to the production of four well-known MonAzPs: two yellow pigments (monascin **1** and ankaflavin **3**) and two orange pigments (rubropunctatin **2** and monascorubrin **4**, Figure 4). Compared to the wild-type strain, no gross differences in colony growth, morphology, or coloration were observed upon the deletion of the *mrpigAup1* and *mrpigAup2* genes at the “left” border, and with the knockout of the *mrpigPdown1* and *mrpigPdown2* genes at the “right” border. The production of MonAzPs was also unchanged with these strains, with the yields and mutual ratios of compounds **1**, **2**, **3,** and **4** indistinguishable from those of the wild-type strain (Figure 4). These results confirm that *mrpigAup2* and *mrpigPdown2* are not part of the MonAzPs BGC, in agreement with the comparative genomic and transcriptional analyses (Figures 1, 2). However, these results also show that *mrpigAup1* and *mrpigPdown1* do not play discernible roles in MonAzPs biosynthesis and thus should not be considered to be part of the MonAzPs BGC, in spite of the contrary prediction from the transcriptional analysis (Figure 2).

In contrast, the Δ*mrpigA* and Δ*mrpigO* strains exhibited significantly different phenotypes and MonAzPs production profiles, compared to that of *M. ruber* M7. The colonies of Δ*mrpigA*, with the deletion affecting this gene at the “left” border of the cluster, were off-white with the production of all MonAzPs completely abolished. This is in agreement with the crucial role of the MrPigA PKS in the formation of the polyketide core of all MoAzPs in this fungus. The colonies of the Δ*mrpigO* strain, with the knockout eliminating the second gene from the “right” border of the predicted BGC, were yellow to pale orange. This strain produced two known MonAzPs derailment products, the yellow pigments acetyl-monasfluol A and B (compounds **5** and **6**; Chen et al., 2019, Figure 4C). This is consistent with MrPigO being a deacetylase, and emphasizes the central role of this enzyme in favoring the appropriate intramolecular Knoevenagel cyclization that yields MonAzPs congeners with the characteristic linear tricyclic ring system, instead of the angular system seen in shunt products **5** and **6** (Chen et al., 2019). The phenotype of the Δ*mrpigP* strain, with the deletion affecting this gene at the “right” border of the cluster, was similar to that of *M. ruber* M7. The Δ*mrpigP* strain produced large amounts of MonAzPs, although with a shift in the product ratios toward monascin **1** and ankaflavin **3**. MrPigP is an MFS transporter with a role in the export of monasones, the naphthoquinone co-metabolites of this supercluster (Li et al., 2020). However, deletion of this gene was seen earlier not to influence MonAzPs production in liquid media (Li et al., 2020), so this transporter plays, at best, a supplementary part in azaphilone pigment production.

Taken together, our comparative genomic, differential transcriptomic, and gene knockout data predict that the MonAzPs BGC of *M. ruber* M7 extends from *mrpigA* to *mrpigP*, with *mrpigAup1*, *mrpigAup2*, *mrpigPdown1*, and *mrpigPdown2* laying just outside the borders of the cluster.

### Bioinformatic Analysis of the Azaphilone Gene Cluster in *T. marneffei*

The gene cluster in *T. marneffei* ATCC 18224 that we took advantage of to predict the boundaries of the MonAzPs BGC in *M. ruber* M7 is nearly identical to a genomic locus in *T. marneffei* PM1. This locus in strain PM1 was described to contain a 5–gene BGC responsible for the production of a variety of soluble red-colored *Monascus-*type azaphilone pigments (Woo et al., 2014). These compounds are γ-vinylogous pyridines that are produced when the orange azaphilone pigments, rubropunctatin **2,** and monascorubrin **4** form adducts to effect an *O*-to-*N* substitution with various amines, including amino acids from the cell or the media. The same BGC was also described to be responsible for the biosynthesis of yellow azaphilone pigments such as ankaflavin **3** in strain PM1, as expected (Woo et al., 2014).

Comparison of the *M. ruber* M7 MonAzPs BGC with the genome sequences of *T. marneffei* ATCC 18224 and PM1 showed that most MonAzPs biosynthetic genes are conserved and localized in a single locus in the two *Talaromyces* genomes (Supplementary Table 4), although these pigment BGCs in *Monascus* vs. the two *T. marneffei* strains are not syntenic. Genes similar to *mrpigF* and *mrpigO* of *M. ruber* M7 are encoded outside of the *T. marneffei* BGCs, while orthologs of the ankyrin repeat protein-encoding *mrpigL* are not immediately obvious, nor are they necessary for azaphilone pigment biosynthesis.

We propose that the apparent *Monascus-*type azaphilone BGC in *T. marneffei* ATCC 18224 includes the genes XP_002149758 to XP_002149772 (corresponding to KFX40792 to KFX45538 in strain PM1, Supplementary Table 4). Together with the additional MonAzPs gene homologs that are outside the BGCs in the *Talaromyces* genomes, this cluster is the functional equivalent of the MonAzPs BGC of *M. ruber* M7 (Supplementary Table 4, Woo et al., 2014; Chen et al., 2019). Importantly, our model for the *Talaromyces* BGCs includes a much more extensive genomic region than the originally delineated five-gene cluster (Woo et al., 2014), again highlighting the difficulties of boundary predictions for complex BGCs in fungi. Functional verification of this more extensive BGC using transcriptomic and gene knockout studies is still necessary to clarify the biosynthesis of *Monascus-*type azaphilone pigments in the two *Talaromyces* strains.

## Discussion

A significant aspect of fungal development is the production of SMs that serve as allelochemicals, pigments, intra- and interspecies signaling compounds and modulators of metabolic processes. Many of these molecules display a broad range of antibiotic, antitumor, enzyme modulatory, and immunosuppressive activities that can be exploited for drug discovery and development (Hyde et al., 2019). Advances in genomics revealed that the genes necessary for the production of a given SM are typically (although not always) clustered on the fungal genome (Weber et al., 2009). Such BGCs encode core enzymes for SM scaffold biosynthesis; modifying enzymes for scaffold editing, complexity generation, and SM maturation; and transcription factors, self-resistance, and SM export mediators (Keller, 2019).

Traditionally, SM BGCs are detected in genome sequence assemblies by identifying the core genes (such as PKSs, NRPSs, or terpene synthases), based on their conserved sequence motifs. Next, the putative BGCs are extended by recruiting flanking genes that are similar to genes frequently found in known SM BGCs, including those that encode hydroxylases, oxidases, methyltransferases, transcription factors, and transporters (Keller, 2019). This process is often facilitated by specialized software tools, such as SMURF, antiSMASH, CASSIS, or Prism, and may utilize curated repositories such as MIBiG (Khaldi et al., 2010; Wolf et al., 2016; Blin et al., 2019; Kautsar et al., 2020; Skinnider et al., 2020). While this workflow met with spectacular success in the last two decades (Khaldi and Wolfe, 2011; Wiemann et al., 2013b; Leadmon et al., 2020), SM BGCs without core genes remain difficult to detect, and cluster boundaries usually remain provisional or even arbitrary. The MonAzPs BGC is a prime example for the latter problem: different prediction methods and different authors delimited this cluster with widely different boundaries (Figure 2A), despite the availability of multiple genome sequences from several MonAzPs-producing fungi, mostly *Monascus* spp.

We decided to investigate this problem by cross-referencing a motif-independent, comparative genomics-based BGC prediction method (Takeda et al., 2014) with differential transcriptional analysis in a cluster-specific regulator mutant, and gene knockouts followed by the analysis of MonAzPs production by the resulting strains. These approaches collectively defined the MonAzPs BGC of *M. ruber* M7 to extend from *mrpigA* to *mrpigP*, encompassing 16 genes. This assignment, supported by multiple lines of experimental evidence, offers a more secure basis for the retrobiosynthetic analysis of MonAzPs production, and provides a better focus for engineering approaches toward designer *Monascus-*type azaphilone pigments that may be useful to produce nutraceuticals, food and feed products, and specialty chemicals (Tolborg et al., 2020). Additional transcriptional studies utilizing different cultivation conditions may further refine BGC border assignments.

The BGC responsible for MonAzPs production in *Talaromyces (Penicillium) marneffei* ATCC 18224 was also provisionally delimited during our comparative genomics process (Figure 1). *T. marneffei* was reported to produce MonAzPs-type soluble red pigments (Ogihara et al., 2000, 2001; Frisvad et al., 2013; Arai et al., 2015; Jin et al., 2018; Morales-Oyervides et al., 2020; Tolborg et al., 2020), and accordingly, orthologs of most of the *M. ruber* M7 MonAzPs biosynthetic genes were found to be encoded in the *T. marneffei* ATCC 18224 BGC, albeit in a non-syntenic arrangement (Chen et al., 2017, 2019; Pavesi et al., 2021). While the genes corresponding to *mrpigF*, *mrpigO*, and *mrpigL* were absent from the *T. marneffei* BGC, similar genes to *mrpigF* and *mrpigO* are encoded elsewhere in the genome of this fungus (Supplementary Table 4), and a *mrpigL* equivalent is not necessary for azaphilone pigment biosynthesis. Functional studies with a near-identical genomic locus in *T. marneffei* strain PM1 have been reported by Woo et al. (2014). Surprisingly, the knockdown of only five genes of this locus was shown to lead to the loss of *Monascus-*type azaphilone pigment production. Correspondingly, only *pks3* (66% identity to *mrpigA*), *rp1* (46% identity to *mrpigB*), *rp2* (58% identity to *mrpigK*), *rp3* (58% identity to *mrpigJ*), and *rp4* (65% identity to *mrpigH*) were annotated as constituents of the *Monascus-*type azaphilone BGC in *T. marneffei* PM1. Further research is necessary to reveal why the knockdown of genes *orf3* (65% identity to *mrpigN*), *orf4* (63% identity to *mrpigD*), *orf5* (70% identity to *mrpigC*), *orf7* (71% identity to *mrpigG*), *orf8* (70% identity to *mrpigE*), or *orf9* (57% identity to *mrpigP*) were seen to be neutral for pigment biosynthesis in *T. marneffei* PM1 when the knockout of the orthologous genes in *M. ruber* M7 eliminates or at least severely reduces the production of the classical MonAzPs **1–4** (Liu et al., 2014; Woo et al., 2014; Chen et al., 2017). In addition, gene knockdown experiments in *T. marneffei* PM1, targeting *pks3* (XP_002149769 in strain ATCC 18224, the ortholog of the *mrpigA* PKS), and *rp1* (XP_002149768 in strain ATCC 18224, the ortholog of *mrpigB*, encoding a transcriptional regulator) seem to also connect the BGC in strain PM1 to the production of citrinin, a mycotoxin with a similar but not identical chromophore to that of MonAzPs (Woo et al., 2014). Importantly, citrinin biosynthesis is governed in *Monascus* spp. by a BGC different from the one for MonAzPs (He and Cox, 2016), necessitating the further evaluation of the proposed connection of the *T. marneffei* PM1 BGC (and its equivalent in strain ATCC 18224) to citrinin biosynthesis. Integration of further bioinformatic, transcriptomic, gene knockdown/gene knockout, gene overexpression, and metabolite analyses, as described here for the MonAzPs BGC of *M. ruber* M7, is expected to answer such outstanding questions.

The MonAzPs BGC represents an especially tough challenge for cluster boundary prediction. This is because of the composite, mosaic-like nature of this BGC. First, it is a supercluster with genes necessary to produce two structurally different SM groups: MonAzPs-type azaphilones and monasone-type naphthoquinones (Li et al., 2020). Some genes are involved solely in the biosynthesis of one or the other SM group, while others are necessary for both. Next, MonAzPs production itself is highly complex, with many metabolic branch points opening up shunt pathways, and integrating fortuitous enzymatic and chemical reactions that afford a huge variety of pigment products (Chen et al., 2019). Last, this BGC also straddles genes or even sub-clusters (such as the six-gene sub-cluster in *M. pilosus* and *M. ruber* NRRL 1597) with no relationship to the production of MonAzPs or monasones (Balakrishnan et al., 2013; Chen et al., 2017). Any and all of these characteristics could easily confuse BGC border prediction methods that rely on one type of dataset. Thus, during the trimming step of the comparative genomics-based prediction (Takeda et al., 2014), the calculated scores at *mrpigG*, *mrpigK*, and *mrpigN* all showed inflection points (Figure 1D), potentially misleading a user to call the end of the MonAzPs BGC at any of these genes. When analyzing differential trascriptomic data by comparing the wild-type and the Δ*mrpigB* strains, the expression levels of the *mrpigC*, *mrpigJ*, *mrpigK*, or *mrpigL* genes were not significantly downregulated in the strain that is deficient in the cluster-specific regulator (Figure 2B). This could have been taken as a sure sign that the end of the BGC was already reached. Conversely, although both the *mrpigAup1* (at the left border) and the *mrpigPdown1* gene (at the right border) were found to be downregulated in Δ*mrpigB* strains (Figure 2B), neither comparative genomics nor gene knockouts support the inclusion of these genes in the MonAzPs BGC (Figures 1, 4). Finally, gene disruption of *mrpigL*, a gene well within the BGC boundaries, does not affect the production of MonAzPs (Chen et al., 2017), again giving a false signal for reaching the end of the BGC. Based on these and similar examples, we believe that only the integration of all three methods used in this study (comparative genomics, differential transcriptomics, and gene deletion with subsequent metabolite analysis) could adequately identify BGC borders in fungi, at least in the case of highly complex clusters such as the one for MonAzPs production.

## Data Availability Statement

The datasets presented in this study can be found in online repositories. The names of the repository/repositories and accession number(s) can be found in the article/Supplementary Material.

## Author Contributions

QL designed and performed the experiment, contributed to data analysis, and wrote the draft manuscript. SZ, XW, and SG contributed to bioinformatic analysis. XY and FC contributed to manuscript editing. IM designed the project, analyzed the data, and wrote and edited the manuscript. All authors have read and approved the manuscript prior to submission.

## Conflict of Interest

IM has disclosed financial interests in Teva Pharmaceutical Works Ltd., Hungary and the University of Debrecen, Hungary that are unrelated to the subject of the research presented here. The remaining authors declare that the research was conducted in the absence of any commercial or financial relationships that could be construed as a potential conflict of interest.

## Footnotes

1 www.jcvi.org/smurf/

2 http://antismash.secondarymetabolites.org

3 http://www.broadinstitute.org/scientific-community/data

4 http://www.ncbi.nlm.nih.gov/genome/

5 http://smurf.jcvi.org

6 https://sbi.hki-jena.de/cassis/

7 http://linux1.softberry.com/berry.phtml

8 http://pfam.sanger.ac.uk/

9 http://blast.ncbi.nlm.nih.gov/Blast.cgi

10 https://prism.adapsyn.com/
