# Supplementary material for: An Integrated Approach to Determine the Boundaries of the Azaphilone Pigment Biosynthetic Gene Cluster of Monascus ruber M7 Grown on Potato Dextrose Agar
Source: Front Microbiol. 2021 Jun 16;12:680629. doi: 10.3389/fmicb.2021.680629 (PMC8241920; doi:10.3389/fmicb.2021.680629)
Supplement: Supplementary file 1 [file Data_Sheet_1.docx]

Supplementary Material for *Frontiers in Microbiology*.

**An integrated approach to determine the boundaries of the azaphilone pigment biosynthetic gene cluster of *Monascus ruber* M7 grown on potato dextrose agar**

Qingpei Liu, ^1,2^ Siyu Zhong, ^1^ Xinrui Wang, ^1^ Shuaibiao Gao,^1^ Xiaolong Yang, ^1^*** Fusheng Chen, ^3,4^*** and István Molnár ^2^***

^1^ The Modernization Engineering Technology Research Center of Ethnic Minority Medicine of Hubei Province, School of Pharmaceutical Sciences, South-Central University for Nationalities, Wuhan, Hubei Province, 430074, P.R. China

^2^ Southwest Center for Natural Products Research, The University of Arizona, 250 E. Valencia Rd., Tucson, Arizona, 85706, U.S.A.

^3^ Hubei International Scientific and Technological Cooperation Base of Traditional Fermented Foods, Huazhong Agricultural University, Wuhan, Hubei Province, 430070, P.R. China

^4^ College of Food Science and Technology, Huazhong Agricultural University, Wuhan, Hubei Province, 430070, P.R. China

* Corresponding authors: Xiaolong Yang, E-mail: [yxl19830915@163.com](mailto:yxl19830915@163.com); Fusheng Chen, E-mail: [chenfs@mail.hzau.edu.cn](mailto:chenfs@mail.hzau.edu.cn); and István Molnár, E-mail: [imolnar@email.arizona.edu](mailto:imolnar@email.arizona.edu)

Contents

[Table S1. Genome sequence assemblies used for comparative genomics S1](#_Toc68626679)

[Table S2. Primers for the qRT-PCR analysis of the MonAzPs BGC S2](#_Toc68626680)

[Table S3. Primers used for the gene knockouts S3](#_Toc68626681)

[Table S4. Comparison of MonAzPs BGCs from *M.* *ruber* M7, *T. marneffei* ATCC 18224, and *T. marneffei* PM1 S4](#_Toc68626682)

[Figure S1. Knockout of the *mrpigAup1* gene in *M. ruber* M7 S5](#_Toc68626683)

[Figure S2. Knockout of the *mrpigAup2* gene in *M. ruber* M7 S6](#_Toc68626684)

[Figure S3. Knockout of the *mrpigPdown1* gene in *M. ruber* M7 S7](#_Toc68626685)

# Table S1. Genome sequence assemblies used for comparative genomics

| Species | Genome size (Mb) | Source |
| --- | --- | --- |
| *Aspergillus nidulans* FGSC A4 | 30.07 | The Broad Institute *Aspergillus* comparative database (https://www.broadinstitute.org/fungal-genome-initiative/aspergillus-genome-projects) |
| *A.* *niger* ATCC 1015 | 37.20 |  |
| *A. flavus* NRRL 3357 | 36.79 |  |
| *A. oryzae* RIB40 | 37.12 |  |
| *A. terreus* NIH 2624 | 29.33 |  |
| *A. fumigatus* Af293 | 29.38 |  |
| *Neosartorya fischeri* NRRL 181 | 32.55 |  |
| *Talaromyces marneffei* ATCC 18224 | 28.64 | GenBank: ABAR00000000.1 |
| *Monascus ruber* M7^a^ | 23.81 | Chen, F. *et al.*, unpublished. |

^a^ *M. ruber* M7 strain: CCAM 070120 (Culture Collection of State Key Laboratory of Agricultural Microbiology, part of the China Center for Type Culture Collection, Wuhan, China

# Table S2. Primers for the qRT-PCR analysis of the MonAzPs BGC

| **Name** | **Sequence (5′→3′)** | **PCR product (bp)** |
| --- | --- | --- |
| mrpigAup2F | ATTCAAGCATTCGAAGGCAT | 233 |
| mrpigAup2R | CATCTCCTTTTCCACGGCT |  |
| mrpigAup1F | AAGCACGACCGACCTTTCCT | 278 |
| mrpigAup1R | GTGTCACCAGTGGCATCAGC |  |
| mrpigAF | GTCATTGGCATGTCGTGTAAGG | 182 |
| mrpigAR | GCATCGTGGTCTCGGATAAAG |  |
| mrpigBF | CGACCTCGATACCCGAACTGA | 129 |
| mrpigBR | CCTCGTTCTTGTGGGCATCTT |  |
| mrpigCF | CGCATCTTCTCAACCCGACT | 146 |
| mrpigCR | CATGATGTCCGTAGGGCAATT |  |
| mrpigDF | CGTCGTCTCGCCCGATAACT | 272 |
| mrpigDR | CCATCACGCCCATGTACTCC |  |
| mrpigEF | CGCTACTGGAACGATGCCTAC | 157 |
| mrpigER | CACTGCCACAGACCAAGATGG |  |
| mrpigFF | GGCAACCATACTCCGACTCAT | 100 |
| mrpigFR | ACCACGTATTCCGGTAACCCT |  |
| mrpigGF | CGGCATGTCTCACGTCTCCT | 244 |
| mrpigGR | TCCTTCGTCTTGATCGGCAC |  |
| mrpigHF | CCAGGGCCGCAAGTTTATC | 186 |
| mrpigHR | CCGATGCCGTTGTGATTGA |  |
| mrpigIF | ATGCGGCAAGTCGGAAACAT | 93 |
| mrpigIR | GGCGGCAGAAGCCATTGTAG |  |
| mrpigJF | CGCCCGTAAAGTTCGTCAGTA | 284 |
| mrpigJR | GCGTGGTGAATCGGTAGACA |  |
| mrpigKF | GACCAGACTCGCATACCACATA | 141 |
| mrpigKR | GACAACCCTCCAAGGAAATAGA |  |
| mrpigLF | TACGGCACGGAACCAGAACC | 124 |
| mrpigLR | AATTCGGCAAACATCATCAACAT |  |
| mrpigMF | CGAATACCCTCCCATCCAATC | 104 |
| mrpigMR | AGTGACGCTGATCCGACGAT |  |
| mrpigNF | TCCAGCTTGGCGAAGACC | 162 |
| mrpigNR | GCATTTGGCAGCATCACG |  |
| mrpigOF | GCACAAAGCCTGGAAGACGAT | 274 |
| mrpigOR | AAGCTGCATTGCACCGAAC |  |
| mrpigPF | TCTGTTGTCATCGGGCTCTTC | 318 |
| mrpigPR | GGGCAGGTAGTAACCAAATCG |  |
| mrpigPdown1F | CCCTGGTATCATGGGCTGTG | 191 |
| mrpigPdown1R | TGGTCCTGGCTCCCTCTTTT |  |
| mrpigPdown2F | TGGAAGGGCTGGCTAATGA | 188 |
| mrpigPdown2R | TGGACCTTGCCATCTTCTGT |  |
| GADPHF | CAAGCTCACTGGCATGTCTATG | 243 |
| GADPHR | AAGTTCGAGTTGAGGGCGATA |  |

# Table S3. Primers used for the gene knockouts

| **Name** | **Sequence (5′→3′)** | **Description of the amplicon (length)** |
| --- | --- | --- |
| P1 | GTCCACCACTCATACATT | 5′ flanking region of the *mrpigAup1* gene (910 bp) |
| P2 | TCCTTCAATATCATCTTCTGTCGAC  ATCTATCGCTTCTTCCTAG |  |
| P3 | GTTTAGAGGTAATCCTTCTTTCTAG  CAAGGTACACAGTACTTGAG | 3′ flanking region of the *mrpigAup1* gene (893 bp) |
| P4 | GATGGGTTTCCTGCACTGAAT |  |
| P5 | AGCTGGTCGGATTATCTTTGAG | Internal segment of the *mrpigAup1* gene (1,053 bp) |
| P6 | CCTTGTCCGCATCCTTAGCATT |  |
| P7 | GTCGACAGAAGATGATATTG | The *hph* cassette from plasmid pSKH (2,137 bp) |
| P8 | CTAGAAAGAAGGATTACCTC |  |
| P9 | TGTATGAGTGGTGGACAACT | 5′ flanking region of the *mrpigAup2* gene (955 bp) |
| P10 | TCCTTCAATATCATCTTCTGTCGAC  CCTCGATCTTCCGTTGGATCT |  |
| P11 | AGAGGTAATCCTTCTTTCTAG  CCGATTCTTTGACTATGAG | 3′ flanking region of the *mrpigAup2* gene (954 bp) |
| P12 | CGTATTGGCTTGTTGATTTGAC |  |
| P13 | AGCGTCACAACAGCACAG | Internal segment of the *mrpigAup2* gene (658 bp) |
| P14 | ATTCTTCGGTGATCGTTTTGGG |  |
| P15 | AGTTAGGGTTAGCCACAGC | 5′ flanking region of the *mrpigPdown1* gene (898 bp) |
| P16 | TCCTTCAATATCATCTTCTGTCGAC  CAAGGCCGACAAATTCAACG |  |
| P17 | GTTTAGAGGTAATCCTTCTTTCTAG  CTTACGGCGGAATGAGAT | 3′ flanking region of the *mrpigPdown1* gene (716 bp) |
| P18 | TAGCCAGCCCTTCCATCCC |  |
| P19 | GGCAGCTAGTTCTCACAAG | Internal segment of the *mrpigPdown1* gene (2,028 bp) |
| P20 | GCGTTACATGAGTTAGGAT |  |
| P21 | AAAGAACACCCATGCCGGAAGT | 5′ flanking region of the *mrpigPdown2* gene (869 bp) |
| P22 | TCCTTCAATATCATCTTCTGTCGAC  AGGCTATCTACGCGCACGAAAT |  |
| P23 | GTTTAGAGGTAATCCTTCTTTCTAG  TCGGGACTGGAAGTCTACTCAG | 3′ flanking region of the *mrpigPdown2* gene (972 bp) |
| P24 | CGGAAGATGGCTACGCTATTG |  |
| P25 | CGGGCAACGATGTCAATAAG | Internal segment of the *mrpigPdown2* gene (1,153 bp) |
| P26 | TAGACTTCCAGTCCCGACCAAC |  |
| P27 | GAGCGCGATCGCAGTTTGAGGT | Knockout cassette outside primers to verify the ∆*mrpigAup1* strain |
| P28 | CGTAAATATATACAGGCCT |  |
| P29 | ATCGAGATCTAGTTACTTTCGT | Knockout cassette outside primers to verify the ∆*mrpigAup2* strain |
| P30 | TCCCCGTACATGGGATTTTG |  |
| P31 | TCCGCGGAGTGGCCTAAAC | Knockout cassette outside primers to verify the ∆*mrpigPdown1* strain |
| P32 | CAGACGCTTCGCCTCGTGGA |  |
| P33 | GTAGAGATTCAGGATGTTCAG | Knockout cassette outside primers to verify the ∆*mrpigPdown2* strain |
| P34 | CGGGCATTCGGCCGCAGAT |  |

# Table S4. Comparison of MonAzPs BGCs from *M.* *ruber* M7, *T. marneffei* ATCC 18224, and *T. marneffei* PM1

| **Protein in**  ***M. ruber* M7** | **GenBank Accession** | **Predicted function** | **Homolog in**  ***T. marneffei* ATCC 18224** | **Amino acid sequence identity (%) with *M. ruber* M7** | **Homolog in**  ***T. marneffei* PM1** | **Amino acid sequence identity (%) with ATCC 18224** |
| --- | --- | --- | --- | --- | --- | --- |
| MrPigAup2 | MW557663 | D-tyrosyl-tRNA (Tyr) deacylase | [XP_002148495](https://www.ncbi.nlm.nih.gov/protein/XP_002149769?report=genbank&log$=protalign&blast_rank=5&RID=AGNPX3DG014) | 66 | KFX51623 | 100 |
| MrPigAup1 | QBY06319 | Peptidyl-prolyl *cis-trans* isomerase Cpr7 | XP_002148500 | 71 | KFX51620 | 100 |
| MrPigA | ALN44200 | Polyketide synthase | [XP_002149769](https://www.ncbi.nlm.nih.gov/protein/XP_002149769?report=genbank&log$=protalign&blast_rank=5&RID=AGNPX3DG014) | 66 | KFX45541 | 99 |
| MrPigB | AGL44390 | Transcription factor | [XP_002149768](https://www.ncbi.nlm.nih.gov/protein/XP_002149769?report=genbank&log$=protalign&blast_rank=5&RID=AGNPX3DG014) | 46 | KFX45543 | 91 |
| MrPigC | ALN44201 | *C*-11-Ketoreductase | [XP_002149770](https://www.ncbi.nlm.nih.gov/protein/XP_002149769?report=genbank&log$=protalign&blast_rank=5&RID=AGNPX3DG014) | 70 | KFX45540 | 100 |
| MrPigD | AGI63864 | 4-*O*-Acyltransferase | [XP_002149771](https://www.ncbi.nlm.nih.gov/protein/XP_002149769?report=genbank&log$=protalign&blast_rank=5&RID=AGNPX3DG014) | 63 | KFX45539 | 100 |
| MrPigE | AHA93896 | NAD(P)H-dependent oxidoreductase | [XP_002149761](https://www.ncbi.nlm.nih.gov/protein/XP_002149769?report=genbank&log$=protalign&blast_rank=5&RID=AGNPX3DG014) | 70 | KFX45563 | 100 |
| MrPigF | APZ73941 | FAD-dependent oxidoreductase | [XP_002145785](https://www.ncbi.nlm.nih.gov/protein/XP_002149769?report=genbank&log$=protalign&blast_rank=5&RID=AGNPX3DG014)^a^ | 41 | KFX44822^a^ | 96 |
| MrPigG | APZ73942 | Serine hydrolase | [XP_002149762](https://www.ncbi.nlm.nih.gov/protein/XP_002149769?report=genbank&log$=protalign&blast_rank=5&RID=AGNPX3DG014) | 71 | KFX45562 | 99 |
| MrPigH | APZ73943 | Enoyl reductase | [XP_002149765](https://www.ncbi.nlm.nih.gov/protein/XP_002149769?report=genbank&log$=protalign&blast_rank=5&RID=AGNPX3DG014) | 65 | KFX45551 | 100 |
| MrPigI | APZ73944 | Transcription factor | [XP_002149764](https://www.ncbi.nlm.nih.gov/protein/XP_002149769?report=genbank&log$=protalign&blast_rank=5&RID=AGNPX3DG014) | 44 | KFX45561 | 81 |
| MrPigJ | AGL44429 | Fatty acid synthase subunit alpha | [XP_002149766](https://www.ncbi.nlm.nih.gov/protein/XP_002149769?report=genbank&log$=protalign&blast_rank=5&RID=AGNPX3DG014) | 58 | KFX45549 | 100 |
| MrPigK | AGL44430 | Fatty acid synthase subunit beta | [XP_002149767](https://www.ncbi.nlm.nih.gov/protein/XP_002149769?report=genbank&log$=protalign&blast_rank=5&RID=AGNPX3DG014) | 58 | KFX45548 | 100 |
| MrPigL | ANS12243 | Ankyrin repeat protein | —^b^ | — | —^b^ | — |
| MrPigM | APZ73945 | *O*-Acetyltransferase | [XP_002149758](https://www.ncbi.nlm.nih.gov/protein/XP_002149769?report=genbank&log$=protalign&blast_rank=5&RID=AGNPX3DG014) | 52 | KFX40792 | 100 |
| MrPigN | ALT31754 | FAD-dependent monooxygenase | [XP_002149772](https://www.ncbi.nlm.nih.gov/protein/XP_002149769?report=genbank&log$=protalign&blast_rank=5&RID=AGNPX3DG014) | 65 | KFX45538 | 100 |
| MrPigO | ANS12244 | Deacetylase | [XP_002149741](https://www.ncbi.nlm.nih.gov/protein/XP_002149769?report=genbank&log$=protalign&blast_rank=5&RID=AGNPX3DG014)^a^ | 41 | KFX46692^a^ | 92 |
| MrPigP | ANS12245 | MFS multidrug transporter | [XP_002149760](https://www.ncbi.nlm.nih.gov/protein/XP_002149769?report=genbank&log$=protalign&blast_rank=5&RID=AGNPX3DG014) | 57 | KFX45564 | 100 |
| MrPigPdown1 | AGI63866 | Formamidase | XP_002153016 | 74 | KFX52887 | 99 |
| MrPigPdown2 | MW557664 | Fructosyl amino acid oxidase | XP_002150918 | 31 | KFX45299 | 100 |

^a^ Genes outside of the MonAzPs BGC in *T. marneffei* ATCC 18224 or PM1

^b^ No homologs found in *T. marneffei* ATCC 18224 or PM1


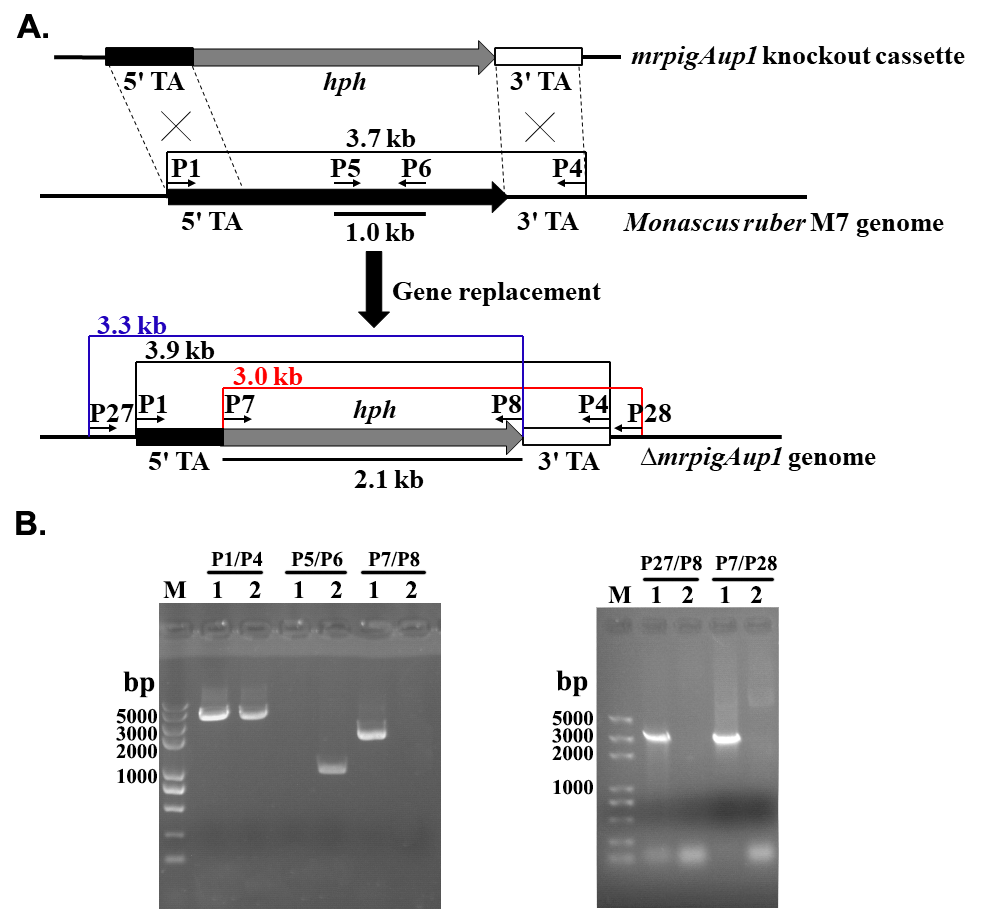


# Figure S1. Knockout of the *mrpigAup1* gene in *M. ruber* M7

**A.** Schematic representation of the gene knockout strategy yielding the Δ*mrpigAup1* strain. The primers, and the sizes of the corresponding PCR amplicons used to verify the gene knockout event are indicated. **B.** Confirmation of the gene deletion event using PCR. *Lane 1*, a representative isolate of the Δ*mrpigAup1* strain; *Lane 2*, the wild-type strain *M. ruber* M7.


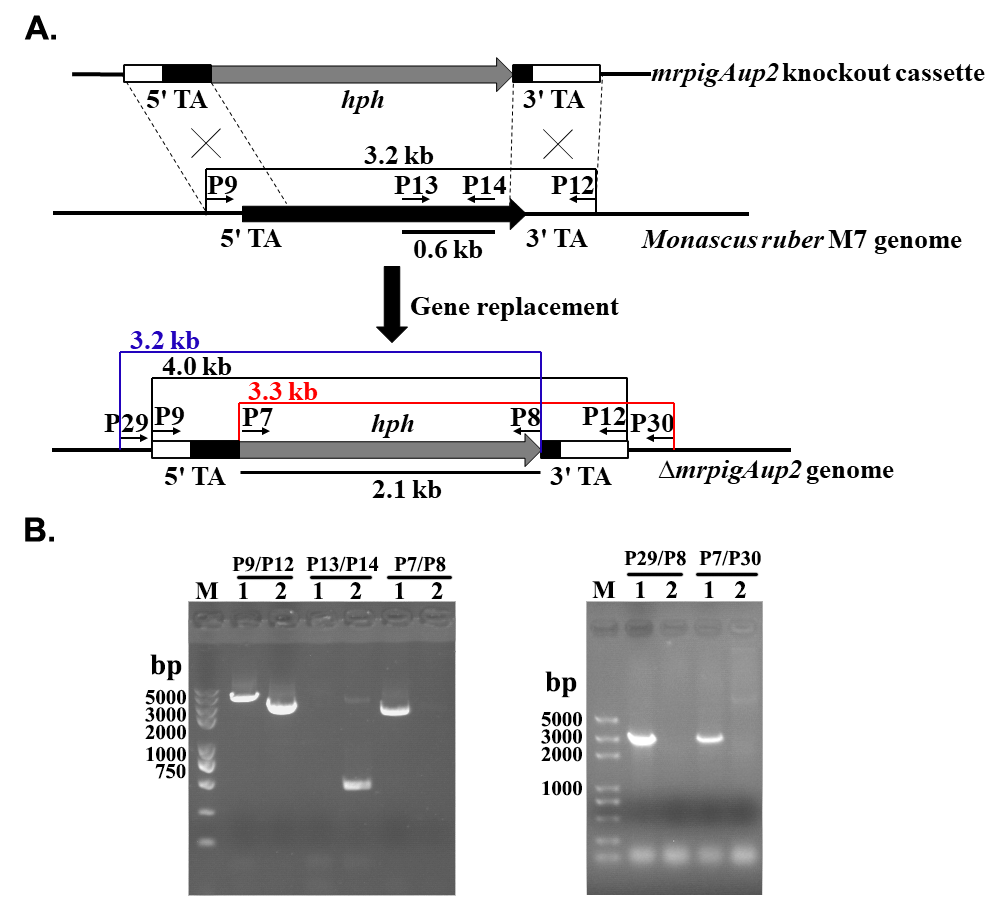


# Figure S2. Knockout of the *mrpigAup2* gene in *M. ruber* M7

**A.** Schematic representation of the gene knockout strategy yielding the Δ*mrpigAup2* strain. The primers and the sizes of the corresponding PCR amplicons used to verify the gene knockout event are indicated. **B.** Confirmation of the gene deletion event using PCR. *Lane 1*, a representative isolate of the Δ*mrpigAup2* strain; *Lane 2*, the wild-type strain *M. ruber* M7.


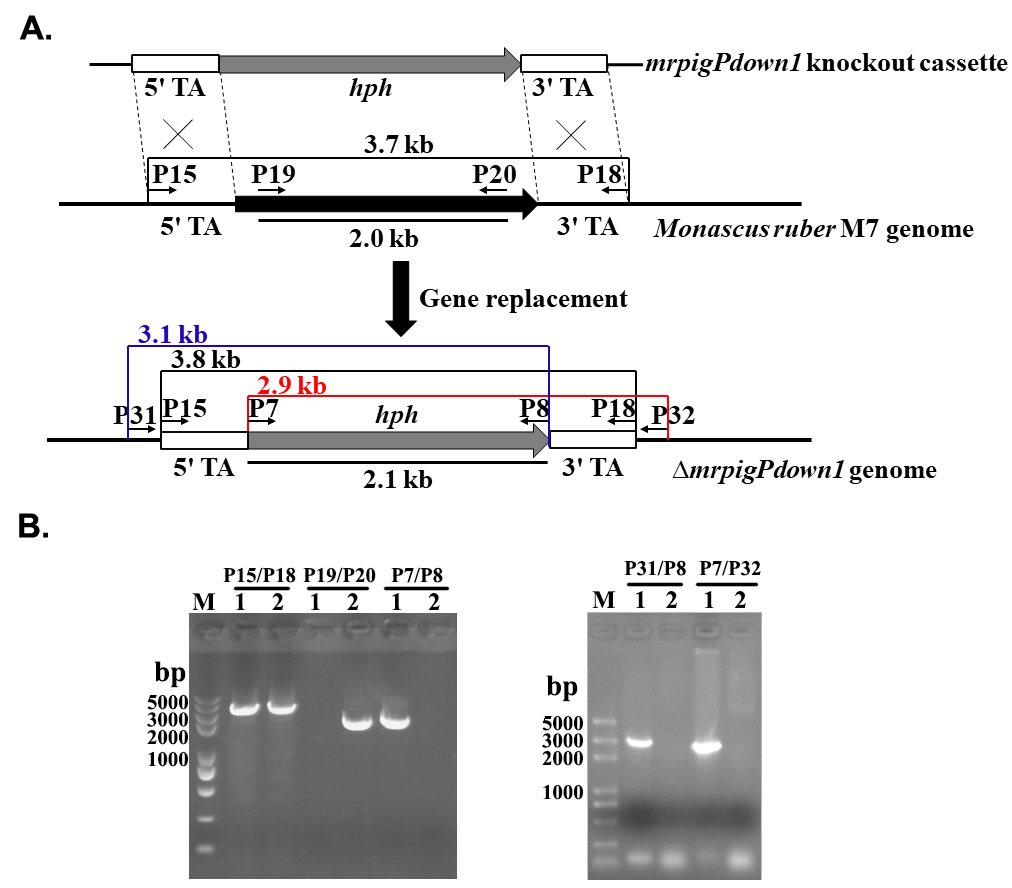


# Figure S3. Knockout of the *mrpigPdown1* gene in *M. ruber* M7

**A.** Schematic representation of the gene knockout strategy yielding the Δ*mrpigPdown1* strain. The primers and the sizes of the corresponding PCR amplicons used to verify the gene deletion event are indicated. **B.** Confirmation of the gene deletion event using PCR. *Lane 1*, a representative isolate of the Δ*mrpigPdown1* strain; *Lane 2*, the wild-type strain *M. ruber* M7.
